# The inner mantle of the giant clam, *Tridacna squamosa*, expresses a basolateral Na^+^/K^+^-ATPase α-subunit, which displays light-dependent gene and protein expression along the shell-facing epithelium

**DOI:** 10.1371/journal.pone.0186865

**Published:** 2017-10-19

**Authors:** Mel V. Boo, Kum C. Hiong, Celine Y. L. Choo, Anh H. Cao-Pham, Wai P. Wong, Shit F. Chew, Yuen K. Ip

**Affiliations:** 1 Department of Biological Sciences, National University of Singapore, Kent Ridge, Singapore, Republic of Singapore; 2 Natural Sciences and Science Education, National Institute of Education, Nanyang Technological University, Singapore, Republic of Singapore; 3 The Tropical Marine Science Institute, National University of Singapore, Kent Ridge, Singapore, Republic of Singapore; Universidade Federal do Rio de Janeiro, BRAZIL

## Abstract

Na^+^/K^+^-ATPase (NKA) is essential for maintaining the Na^+^ and K^+^ gradients, and supporting the secondary active transport of certain ions/molecules, across the plasma membrane of animal cells. This study aimed to clone the *NKA α*-subunit (*NKAα*) from the inner mantle adjacent to the extrapallial fluid of *Tridacna squamosa*, to determine its subcellular localization, and to examine the effects of light exposure on its transcript level and protein abundance. The cDNA coding sequence of *NKAα* from *T*. *squamosa* comprised 3105 bp, encoding 1034 amino acids with an estimated molecular mass of 114 kDa. NKAα had a basolateral localization along the shell-facing epithelium of the inner mantle. Exposure to 12 h of light led to a significantly stronger basolateral NKAα-immunofluorescence at the shell-facing epithelium, indicating that NKA might play a role in light-enhanced calcification in *T*. *squamosa*. After 3 h of light exposure, the transcript level of *NKAα* decreased transiently in the inner mantle, but returned to the control level thereafter. In comparison, the protein abundance of NKAα remained unchanged at hour 3, but became significantly higher than the control after 12 h of light exposure. Hence, the expression of NKAα in the inner mantle of *T*. *squamosa* was light-dependent. It is probable that a higher expression level of NKA was needed in the shell-facing epithelial cells of the inner mantle to cope with a rise in Na^+^ influx, possibly caused by increases in activities of some Na^+^-dependent ion transporters/channels involved in light-enhanced calcification.

## Introduction

Giant clams are marine bivalve mollusks which live in and around coral reefs in the tropical waters of the Indo-Pacific [1]. They live in symbiosis with zooxanthellae (*Symbiodinium*) which are found extracellularly in a branched tubular system embedded in their tissues. With the help of symbiotic zooxanthellae, giant clams can grow at high rates in nutrient deficient tropical waters, but the availability of light critically affects their growth and rate of shell formation [2–4]. The symbiotic zooxanthellae reside mainly inside small tertiary tubules beneath the upper surface of the fleshy and extensible outer mantle [5], where they engage in photosynthesis during insolation. The outer mantle also has iridophores which comprise small groups of cells (iridocytes) containing stacks of tiny reflective platelets [6]. The iridocytes function as a Bragg mirror to scatter light of photosynthetically productive wavelengths into the tissue to benefit the symbionts, and to back-reflect light of non-productive wavelengths [7]. Thus, the extensible outer mantle is brightly colored. By contrast, the inner mantle is in touch with the extrapallial fluid and delineated by the pallial line; it is largely non-pigmented and is involved in shell formation (calcification). Due to the daily cycle of light-enhanced calcification during insolation, the shells of giant clams exhibit striking diurnal variations in the ratio of Sr/Ca in growth bands [8].

Calcification entails the deposition of calcium carbonate through the reaction: Ca^2+^ + HCO_3_^−^ ⇔ CaCO_3_ + H^+^. Hence, the removal of H^+^ would pull the reaction to the right, augmenting the rate of CaCO_3_ precipitation. In fact, the pH of the extrapallial fluid in the fluted giant clam, *Tridacna squamosa*, increases significantly, with a simultaneous decrease in the concentration of ammonia in the extrapallial fluid, during light exposure [9]. It is probable that the H^+^ released during light-enhanced calcification can react with NH_3_ in the extrapallial fluid to form NH_4_^+^. Then, NH_4_^+^ is transported into the shell-facing epithelial cells of the inner mantle, where NH_4_^+^ is turned back into NH_3_ and H^+^, with H^+^ being translocated subsequently into the hemolymph [9]. NH_4_^+^ must be transported from the extrapallial fluid into the epithelial cells of the inner mantle through some sort of active mechanisms, as the total ammonia concentration in the inner mantle is higher than that in the extrapallial fluid [9]. Incidentally, the Na^+^/NH_4_^+^-activated-NKA activity increases significantly in the inner mantle of *T*. *squamosa* exposed to light, and there is also an increase in the effectiveness of NH_4_^+^ to activate NKA by replacing K^+^ [10]. Thus, the transport of NH_4_^+^ from the extrapallial fluid into the epithelial cells of the inner mantle may involve NKA, if it is located at the apical membrane of the shell-facing epithelium. However, NKA has a basolateral localization in nearly all types of epithelial cell [11]; the only exceptions are the choroid plexus [12], the retinal pigment epithelium [13] and the oral epithelium of the coral *Acropora yongei* [14] which express apical NKA.

NKA has three types of subunits (α, β, and γ), and each type of subunit has its isoforms. A functional unit of NKA would comprise minimally one α- and one β-subunit (NKAαβ) [15]. The NKA α-subunit (NKAα; 110–120 kDa) comprises the catalytic domain for binding and transporting of Na^+^ and K^+^, as well as the binding site of ATP [16]. It also contains the specific binding site for ouabain and cardiotonic steroids which can inhibit NKA activity [17]. The NKA β-subunit (NKAβ) is a glycoprotein which facilitates the delivery and insertion of the NKAα into the plasma membrane and contributes to its stability [18] The NKA γ-subunit is not absolutely required for NKA activity, but it modulates NKA function by reducing the affinity of the NKAαβ-complex to Na^+^ and K^+^ and enhancing the complex’s affinity to ATP [19]. Fueled by the hydrolysis of ATP, NKA actively transports 3 Na^+^ out of, and 2 K^+^ (which can be replaced by NH_4_^+^ in some cases) into, the cell. It is pivotal to the maintenance of Na^+^ and K^+^ gradients across the plasma membrane, osmotic balance, and membrane potential in animal cells [20]. It also drives the secondary active transport of ions and molecules such as H^+^, Ca^2+^, HCO_3_^−^, glucose, and amino acids [20]. In epithelial cells, NKA has multiple functions apart from transepithelial ion movements; it is involved in the regulation of structure and function of tight junction, induction of polarity, cell signaling, control of cell movement, and regulation of actin dynamics [21]. These functions appear to be modulated by the enzyme activity of NKA as well as protein–protein interactions of the NKAαβ-complex [16]. There are indications that NKA is involved, albeit indirectly, in biomineralization in the Ca^2+^-transporting sternal epithelium of the terrestrial isopod *Porcellio scaber* [22], the avian eggshell gland [23], scleractinian corals [14,24], and *T*. *squamosa* [10].

As no molecular information on NKA of giant clams is available, this study was performed to clone and characterize the cDNA coding sequence of *NKAα* from the inner mantle of *T*. *squamosa*. This study also aimed to examine mRNA expression level of *NKAα* in the inner mantle in response to light exposure. In addition, a custom-made anti-NKAα antibody was raised commercially to elucidate the subcellular localization of NKAα and to determine the protein abundance of NKAα in the inner mantle. Two hypotheses were tested: (1) the gene and protein expression levels of *NKAα*/NKAα in the inner mantle of *T*. *squamosa* could be affected by light exposure, and (2) NKAα was localized predominantly to the shell-facing epithelium of the inner mantle, which, unlike the sea water-facing epithelium, participated in light-enhanced calcification.

## Materials and methods

### Ethical approval

No institutional (National University of Singapore Institutional Animal Care and Use Committee) approval is required for invertebrates including giant clams at the time the laboratory experiments were performed. The animals were anaesthetized with 0.2% phenoxyethanol before killing to minimize their pain, stress, and suffering.

### Animal

Twenty six individuals of *T*. *squamosa* (average wet mass = 521 ± 184 g) were obtained from XanhTuoi Tropical Fish., Ltd (Vietnam). Maintenance and acclimatization of *T*. *squamosa* were described previously [10,25,26].

### Experimental conditions

At the end of the 12 h dark period, 5 individuals of *T*. *squamosa* exposed to the 12 h light:12 h dark regime were killed for tissue sampling (*N* = 5; control). Separately, tissues were sampled from 5 individuals after 3, 6, or 12 h of exposure to light (*N* = 5 for each time point). Anaesthetization of giant clams was performed with 0.2% phenoxyehtanol prior to tissue sampling. The anaesthetized giant clams were forced open to cut the adductor muscle. The non-pigmented inner mantle was excised, blotted dry and immediately freeze-clamped with aluminium tongs pre-cooled by liquid nitrogen. All samples were stored frozen at -80°C until processing. For immunofluorescence microscopy, six other individuals of *T*. *squamosa* which had been exposed to darkness (*N* = 3) or light for 12 h (*N* = 3) were anaesthetized in 0.2% phenoxyethanol, and their inner mantle tissue from were collected and prepared for immunostaining.

### PCR, RACE PCR and sequencing

Extraction and purification of total RNA from inner mantle were performed as mentioned previously [25,26]. The purified total RNA was quantified by a Shimadzu BioSpec-nanospectrophotometer (Shimadzu Corporation, Tokyo, Japan), checked for integrity by electrophoresis, and converted into cDNA using a RevertAid^™^ first-strand cDNA synthesis kit (Thermo Fisher Scientific Inc., Waltham, MA, USA).

In order to obtain a partial *NKAα* sequence, degenerate primers (Forward: 5’-CTGGTGAYAAMACYGTSATGG-3’ and Reverse: 5’-GAATCATTKACACCATCMCC-3’) were designed using the conserved regions of *Bathypolypus arcticus NKAα* (JN010431.1), *Octopus bimaculatus NKAα* (JN010430.1), *Paroctopus digueti NKAα* (JN010434.1), *Strongylocentrotus purpuratus NKAα isoform X1* (XM_011671680.1) and *S*. *purpuratus NKAα isoform X2* (XM_011671681.1). PCR and cloning experiments were performed following the methods described in Hiong et al. [25,26]. A 9902 Veriti 96-well thermal cycler (Applied Biosystems, Carlsbad, CA, USA) was used to run PCR with DreamTaq^™^ polymerase (Thermo Fisher Scientific Inc.). To obtain the full coding sequence of *NKAα*, 5’ and 3’ RACE were performed with specific primers (Forward: ACCAGAGGAAATTGACCCACATGAGGC and Reverse: ACGCCAAGGAACACAGCTACACCAG) using the SMARTer^™^ RACE cDNA amplification kit (Clontech Laboratories, Mountain View, CA, USA).

Samples were prepared for sequencing using BigDye Terminator v3.1 Cycle Sequencing Kit (Thermo Fisher Scientific) with subsequent ethanol/sodium acetate precipitation. Sequencing was performed using a 3130XL Genetic Analyzer (Thermo Fisher Scientific). BioEdit (version 7.2.5) was employed for sequence assembly and analyses. The *NKAα* sequence obtained for *T*. *squamosa* has been deposited into GenBank (KX858599).

### Deduced amino acid sequence and phenogramic analysis

The ExPASy Proteomic server (http://web.expasy.org/translate/) was used to translate the *NKAα* sequence of *T*. *squamosa* into an amino acid sequence. Transmembrane domains were defined using MEMSAT3 and MEMSAT-SVM provided by the PSIPRED server (http://bioinf.cs.ucl.ac.uk/psipred/). NetPhos 2.0 was employed to predict potential phosphorylation sites. In order to confirm the identity of NKAα, selected amino acid sequences of NKAα from other animals were obtained from Genbank or UniProtKB/TrEMBL and aligned using ClustalX2. Then, a phenogramic analysis was performed using neighbor-joining method and 100 bootstrap replicates with Phylip.

### qPCR

Random hexamer primers with RevertAid^™^ first strand cDNA synthesis kit were used to synthesize cDNA from RNA samples (2 μg) for qPCR analysis. qPCR was performed in triplicates for each sample using a StepOnePlus^™^ Real-Time PCR System (Applied Biosystems), and a set of qPCR primers (forward: 5’-ATGGAATTAGGAGGTCTTGGG-3’; reverse: 5’-TTCACATCATCAGGGTCGT-3’). Each qPCR reaction contained 5 μl of 2x Fast SYBR^®^ Green Master Mix (Applied Biosystems), 0.2 μmol l^-1^ of forward and reverse primers each and various quantities of standard (to construct the standard curve) or 1 ng of sample cDNA in a total volume of 10 μl. The cycling conditions, melt curve analysis and construction of a standard curve were performed according to the method of Hiong et al. [25,26]. The amplification efficiency for *NKAα* was 97.3%. The quantity of *NKAα* transcripts present in a sample was calculated with reference to a standard curve and expressed as number of transcript per ng total RNA.

To substantiate the possible functional relationship between NKA and Na^+^/Ca^2+^ exchanger (NCX) of *T*. *squamosa* in light-enhanced calcification, qPCR primers were also designed for *NCX* expressed in the inner mantle. 0.2 μmol l^-1^ of forward (5’- GACACAATACAGCTCCATCC-3’) and reverse (5’- CTCACCTTGCCTTCATTCTC-3’) primers were used. The amplification efficiency for *NCX* was 97.6%.

### Western blotting

A custom-made anti-NKAα antibody (epitope sequence ELKQELTMDEHKIP) was raised in rabbit by GenScript (Piscataway, NJ, U.S.A.). Protein extraction and SDS-PAGE were performed according to the methods of Hiong et al. [25,26]. Twenty micrograms of proteins from the inner mantle were electrophoretically separated and transferred onto PVDF membranes. Blocking of the membrane was done with 5% skim milk in 1xTTBS (pH 7.6) for 1 h at 25°C. Subsequently the blocked membrane was incubated with the anti- NKAα antibody (1:1000 dilution in TTBS) or the anti-α-tubulin antibody (12G10, 1:20,000 dilution in TTBS) for 1 h at 25°C. The membranes were then incubated in a secondary antibody conjugated with alkaline phosphatase (Santa Cruz Biotechnology Inc.; 1:10,000) diluted in TTBS for 1 h at 25°C. A BCIP/NBT Substrate Kit (Life technologies) was used to visualize the protein of interest. The blots were scanned using a CanoScan 9000F Mark II flatbed scanner in TIFF format at 600 dpi resolution. ImageJ (version 1.50, NIH) was calibrated with a 37-step reflection scanner scale (1″×8″; Stouffer #R3705-1C) before using for the quantification of optical density of bands. The protein abundance was reported as arbitrary optical density of NKAα normalized with that of α-tubulin.

To further support the possible functional relationship between NKA and NCX, Western blotting was also performed using a custom made anti-NCX antibody (epitope sequence: GEDYKPFSEDVTFA) on protein samples obtained from the inner mantle of *T*. *squamosa*.

### Immunofluorescence microscopy

Samples of inner mantle were excised and immersed in 3% paraformaldehyde diluted in seawater at 4°C overnight. Sample preparation and immunostaining were carried out with methods stated in Hiong et al. [26] with the exception that the antigen retrieval was carried out using 0.05% citraconic anhydride with heating at 90°C for 20 min and 1% sodium dodecyl sulfate solution. The concentration of the custom-made anti-NKAα antibody (Genscript) used was 1 μg ml^-1^.

Visualization of the sections was done under an Olympus BX60 epifluorescence microscope (Olympus Corporation, Tokyo, Japan) mounted with an Olympus DP73 digital camera (Olympus Corporation) for image capturing. All images were captured under the optimized exposure settings. NKAα immunostaining was observed using the U-MNIBA filter (Olympus) with excitation at 470–490 nm and 515–550 nm band pass emission filter (green channel). Corresponding differential interference contrast (DIC) images were captured for tissue orientation.

Quantification of fluorescence intensities were performed on original images captured at 400× magnification for shell-facing inner mantle epithelium of giant clams kept in darkness (control) or exposed to light for 12 h, using Image J version 1.50i software with an Olympus Viewer Plugin (http://rsbweb.nih.gov.libproxy1.nus.edu.sg/ij/). Images were converted to greyscale. For each shell-facing epithelium, 6 different regions (the summation of which represented at least 50% of the total area) were randomly chosen for measurement. Regions of similar areas adjacent to the basolateral membrane with little fluorescence were selected for background subtraction. The area, integrated density and mean grey value were used to calculate the total fluorescent intensities in both dark and light samples based on the method of Potapova et al. [27]. Results represent the total fluorescence (integrated density) of 6 different regions randomly selected for an image of an individual clam. A total of six individual clams were quantified (*N* = 3 for control kept in darkness and *N* = 3 for clams exposed to 12 h of light).

### Statistical analysis

Results were presented as means ± standard errors of means (S.E.M). SPSS version 21 (IBM Corporation, Armonk, NY, USA) was used to perform statistical analyses. Homogeneity of variance was checked using Levene’s Test. One-way analysis of variance (ANOVA) was used to evaluate differences among means, followed with multiple comparisons of means by Dunnett’s T3 (for unequal variance) or Tukey’s test (for equal variance). The *P* value for statistical significance was set at 0.05.

## Results

### Nucleotide and amino acid sequences, and phenogramic analysis

**Fig 1:**
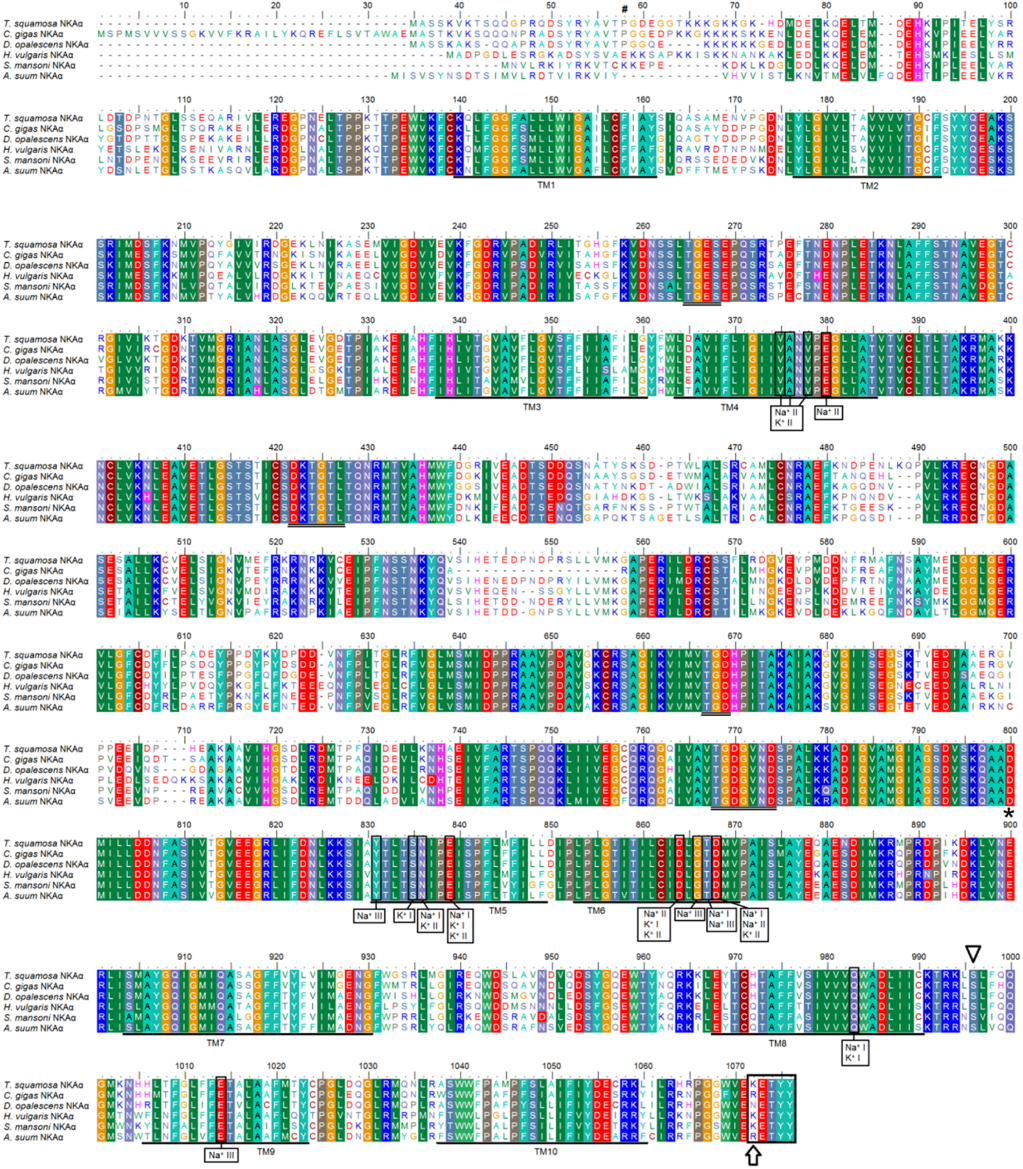
Molecular characterization of Na^+^/K^+^-ATPase α (NKAα) from the inner mantle of *Tridacna squamosa*. Multiple amino acid alignment of NKAα from the inner mantle of *T*. *squamosa*, with other known NKAα from *Crassostrea gigas* (XP_011441273.1), *Doryteuthis opalescens* (ABO61333.1), *Hydra vulgaris* (NP_001296716.1), *Schistosoma mansoni* (CCD78964.1), and *Ascaris suum* (ERG81932.1). Identical or similar amino acid residues are shaded. TM1-TM10 which represents the 10 predicted transmembrane regions are underlined and in bold. Vertical boxes represent coordinating residues for Na^+^ or K^+^ binding. Conserved sequence motifs TGES, PEGL, DKTGT, and GDGVNDSP are double underlined. Amino acid residues which could be phosphorylated by protein kinase A and protein kinase C were denoted with triangle and hash mark, respectively. Asterisk represents the aspartate residue involving in cytoplasmic K^+^ site. A box was used to indicate the KETYY motif and the arrow indicates the replacement of arginine. The transmembrane domains were predicted using MEMSAT3 and MEMSAT-SVM provided by PSIPRED protein structure prediction server.

**Fig 2:**
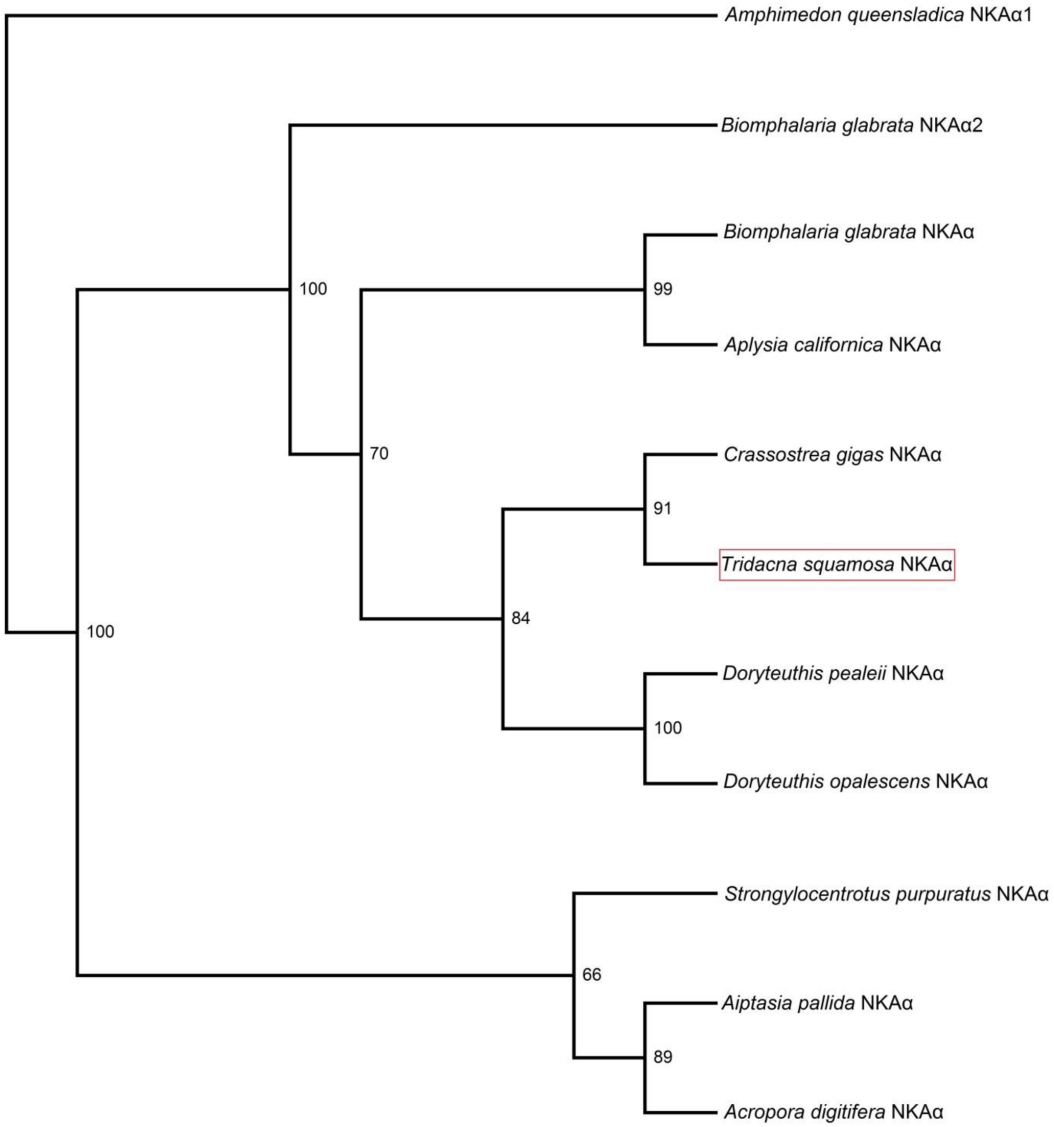
Phenogramic analysis of Na^+^/K^+^-ATPase α (NKAα). A phenogram generated to illustrate the relationship between NKAα from inner mantle of *Tridacna squamosa* and NKAα of selected mollusks, cnidarian, and echinoderm species. Numbers presented at each branch point represent bootstrap values from 100 replicates. NKAα1 of *Amphimedon queensladica* is used as an outgroup.

The complete cDNA coding sequence of the *NKAα* obtained from the inner mantle of *T*. *squamosa* has been deposited into GenBank [Accession: KX858599]. It comprised 3105 bp, encoding 1034 amino acids with an estimated molecular mass of 114.6 kDa (Fig 1). The deduced NKAα sequence of *T*. *squamosa* had 10 predicted transmembrane regions (Fig 1). It comprised conserved regions of NKAα, which include the aspartic acid-lysine-theronine-glycine-threonine (DKTGT) motif containing the phosphorylation site, the proline-glutamic acid-glycine-leucine (PEGL) motif, the threonine-glycine-glutamic acid-serine (TGES) motif, and the glycine-aspartic acid-glycine-valine-asparagine-aspartic acid-serine-proline (GDGVNDSP) motif (Fig 1). A phenogramic analysis confirmed that NKAα of *T*. *squamosa* was closely related to NKAα of other mollusks, cnidarians and echinoderm (Fig 2).

**Fig 3:**
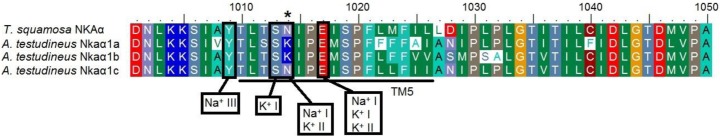
Analyses of Na^+^ and K^+^ binding sites of Na^+^/K^+^-ATPase α (NKAα). A multiple amino acid sequence alignment of a region of NKAα from the inner mantle of *Tridacna squamosa*, with Nkaα1a (JN180940), Nkaα1b (JN180941), and Nkaα1c (JN180942) from the gills of *Anabas testudineus*. Identical amino acid residues are indicated by shaded black residues and similar amino acids (threshold value 50%) are indicated by shaded gray residues. Vertical boxes represent coordinating residues for Na^+^ or K^+^ binding. Asterisks indicate the amino acid residue that is similar to Nkaα1c but different from Nkaα1a and Nkaα1b.

A comparison was made among NKAα of *T*. *squamosa* and Nkaα1a [JN180940], Nkaα1b [JN180941] and Nkaα1c [JN180942] from gills of *Anabas testudineus*. The amino acids constituting the K^+^ binding sites of NKAα of *T*. *squamosa* was identical to those of Nkaα1c (the ammonia-isoform), but different from those of Nkaα1a (the freshwater-isoform) and Nkaα1b (the seawater-isoform) of *A*. *testudineus* (Fig 3).

### Immunofluorescence microscopy

**Fig 4:**
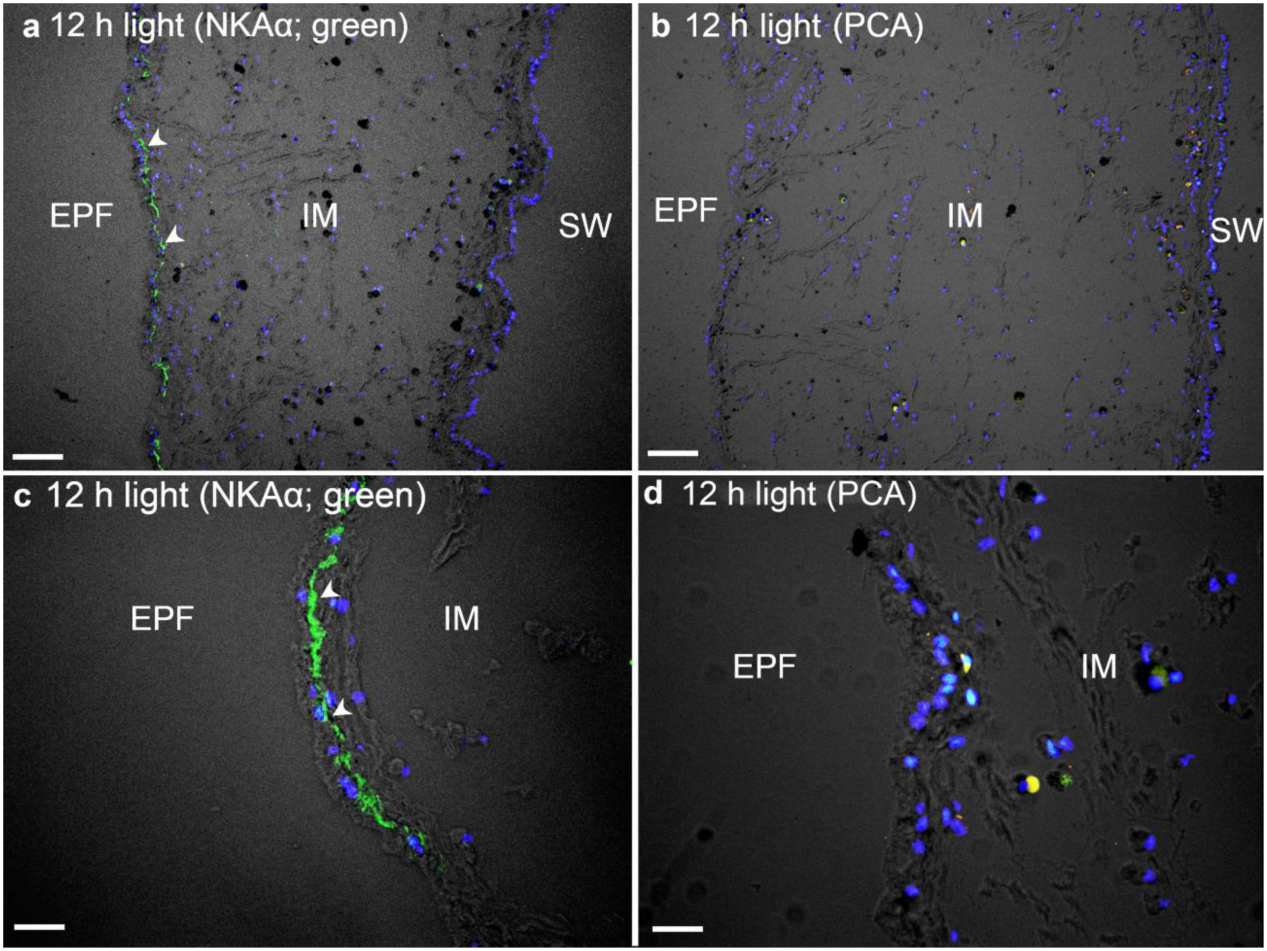
Validation of immunofluorescence of Na^+^/K^+^-ATPase α (NKAα) labelling of the inner mantle of *Tridacna squamosa* by a peptide competition assay (PCA). Immunofluorescent localization of NKAα in the inner mantle (IM) of *T*. *squamosa* exposed to 12 h of light using the custom-made anti-NKAα antibody (A, C), or anti- NKAα antibody pre-incubated with the immunizing peptide in PCA (B, D). Green represents anti-NKAα immunofluorescence. The nuclei are counterstained with DAPI in blue. Together, the green and blue channels are merged and overlaid with differential interference contrast images (DIC). Arrowheads in (A, C) show basolateral staining of NKAα on the epithelium (EP) facing the extrapallial fluid (EPF) compared to the lack of NKAα staining in the control with PCA (b, d). SW, seawater. Scale bar: 20μm.

**Fig 5:**
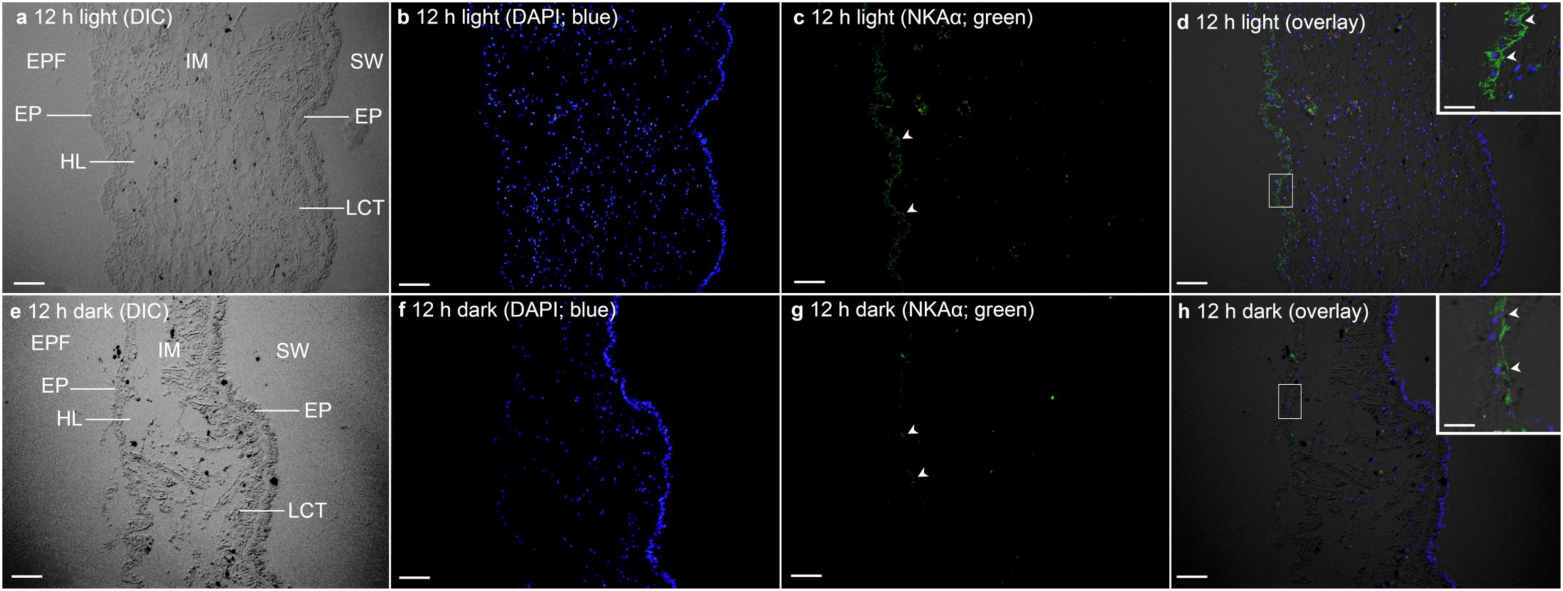
Immunofluorescence microscopy of Na^+^/K^+^-ATPase α (NKAα) in the inner mantle of *Tridacna squamosa*. Immunofluorescent localization of NKAα in the inner mantle (IM) of *T*. *squamosa* exposed to 12 h of light (A to D) or 12 h of darkness (E to H; control). The differential interference contrast images (DIC) labelled with different cellular structures are shown (A, E). The nuclei are counterstained with DAPI in blue (B, F). Anti-NKAα immunofluorescence is displayed in green (C, G). The green and blue channels are merged and overlaid with DIC (D, H). Arrowheads in (C) show more extensive basolateral staining of NKAα on the epithelium (EP) of the IM facing the extrapallial fluid (EPF) as compared to (G). Arrowheads in the insets of (D) and (H) denote more extensive basolateral staining of NKAα on the EP of the IM facing the EPF in (D) compared to (H). No labelling was observed on the EP of the IM facing the seawater (SW) in (C) and (G). HL, hemolymph; LCT, loose connective tissues. Scale bar: 20μm. Reproducible results were obtained from 3 clams exposed to light and 3 clams kept in darkness. Results obtained through quantification of basolateral immunofluorescence of experimental and control clams are reported in the text.

In *T*. *squamosa*, the basolateral membrane of the shell-facing epithelium of the inner mantle was labelled ubiquitously with the anti-NKAα antibody (Fig 4A and 4C). The specificity of the anti-NKAα antibody and the validity of the NKAα-immunolabelling were verified through a blocking peptide competition assay (Fig 4B and 4D). Unlike the shell-facing epithelium (Fig 5C and 5D), the seawater-facing epithelium and the loose connective tissues between the two epithelia displayed very weak immunofluorescence (Fig 5C). The NKAα-immunofluorescence along the basolateral membrane of the shell-facing epithelium of the inner mantle (Fig 5D) of clams exposed to light for 12 h was observed to be greater than that of the control kept in darkness (Fig 5H). Indeed, a quantification (integrated density) of the immunofluorescence of the shell-facing epithelium using ImageJ confirmed that the former was significantly greater (*P*<0.05; 2-fold) than the latter (531 ± 25.7; *N* = 3).

### mRNA expression level and protein abundance of *NKAα*/NKAα

**Fig 6:**
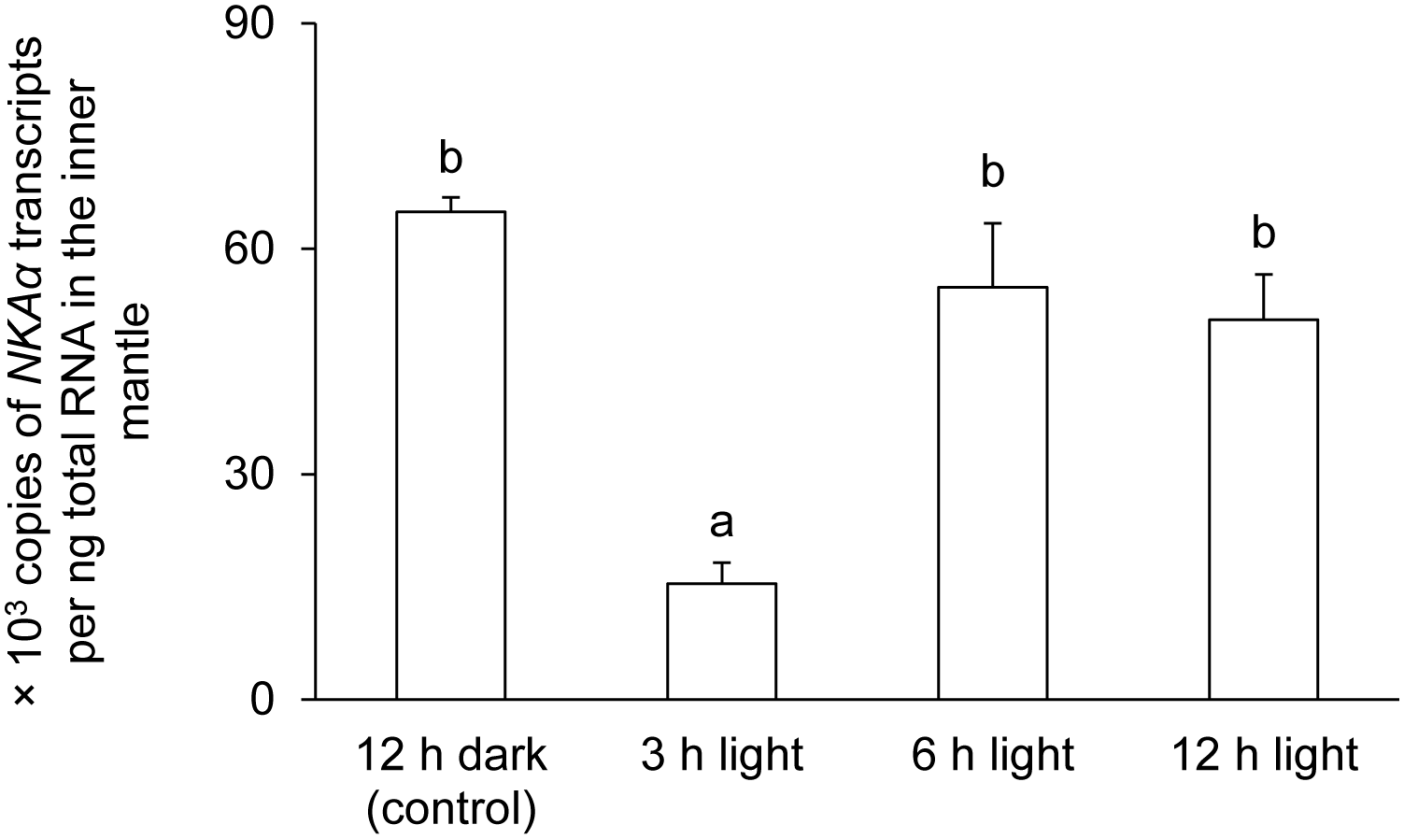
Effects of light on the mRNA expression level of *Na*^+^*/K*^+^*-ATPase α* (*NKAα*) in the inner mantle of *Tridacna squamosa*. The transcript level (x 10^3^ copies of transcripts per ng total RNA) of *NKAα* transcripts in the inner mantle of *T*. *squamosa* exposed to 12 h of darkness (control) or 3, 6 or 12 h of light. Results represent means + S.E.M (*N* = 4). Means not sharing the same letter are significantly different from each other (*P*<0.05).

**Fig 7:**
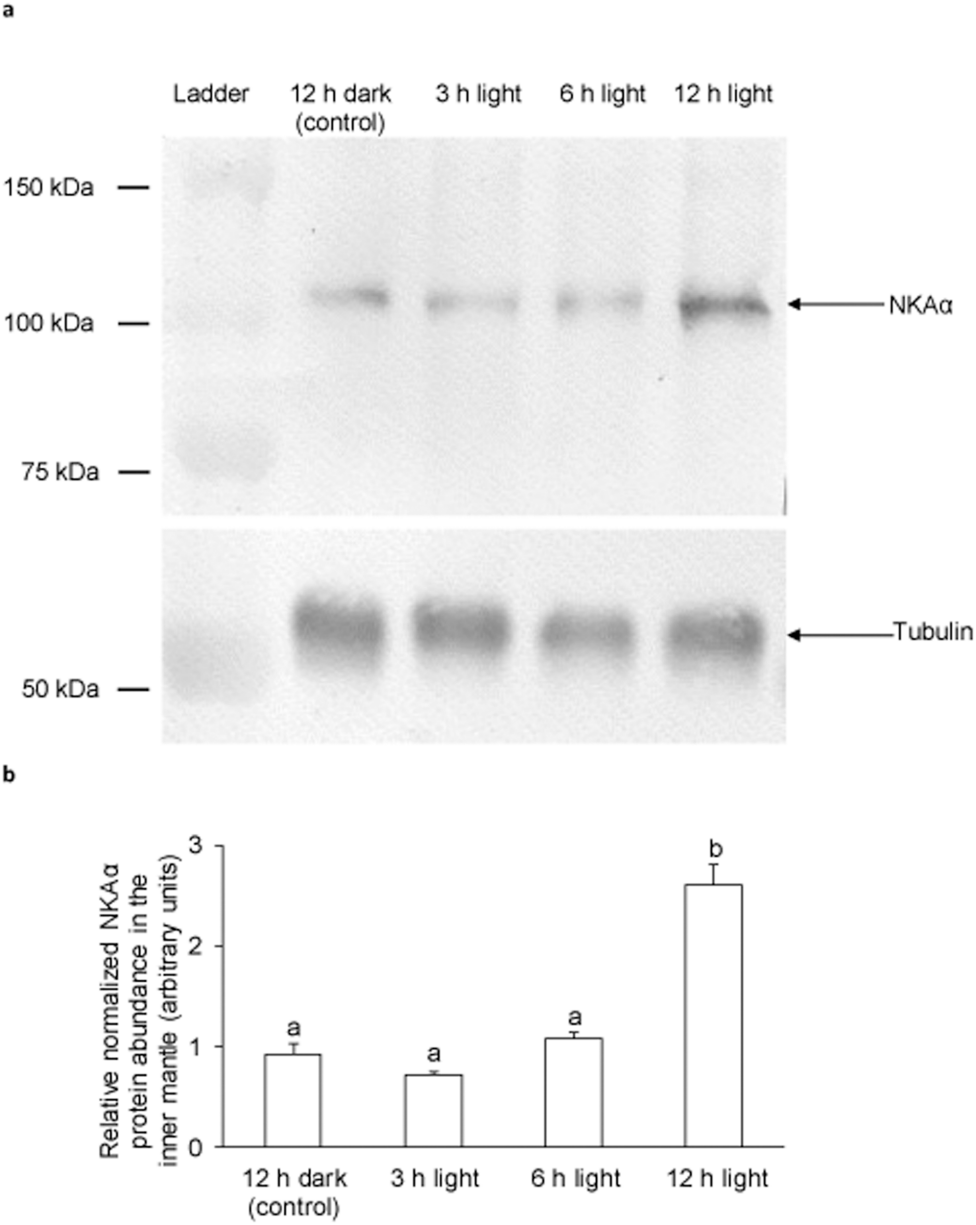
Effects of light on the protein abundance of Na^+^/K^+^-ATPase α (NKAα) in the inner mantle of *Tridacna squamosa*. Protein abundance of NKAα in the inner mantle of *T*. *squamosa* exposed to 12 h of darkness (control) or 3, 6 or 12 h of light. (A) Examples of immunoblot of NKAα, with tubulin as a reference protein. (B) The intensity of the NKAα band for 20 μg protein was normalized with respect to that of tubulin. Results represent means + S.E.M (*N* = 3). Means not sharing the same letter are significantly different (*P*<0.05).

The transcript level of *NKAα* in the inner mantle of *T*. *squamosa* exposed to light for 3 h was significantly lower than that of the control, but it returned back to the control level at 6 h and 12 h of light exposure (Fig 6). Western blotting revealed a band at ~100 kDa, which was close to the deduced molecular mass of NKAα (Fig 7). There were significantly greater (*P*<0.05) protein abundance of NKAα in the inner mantle of *T*. *squamosa* after 12 h of exposure to light as compared with the control (Fig 7), corroborating the qualitative and quantitative results obtained by immunofluorescence microscopy.

### mRNA expression level and protein abundance of *NCX*/NCX

**Fig 8:**
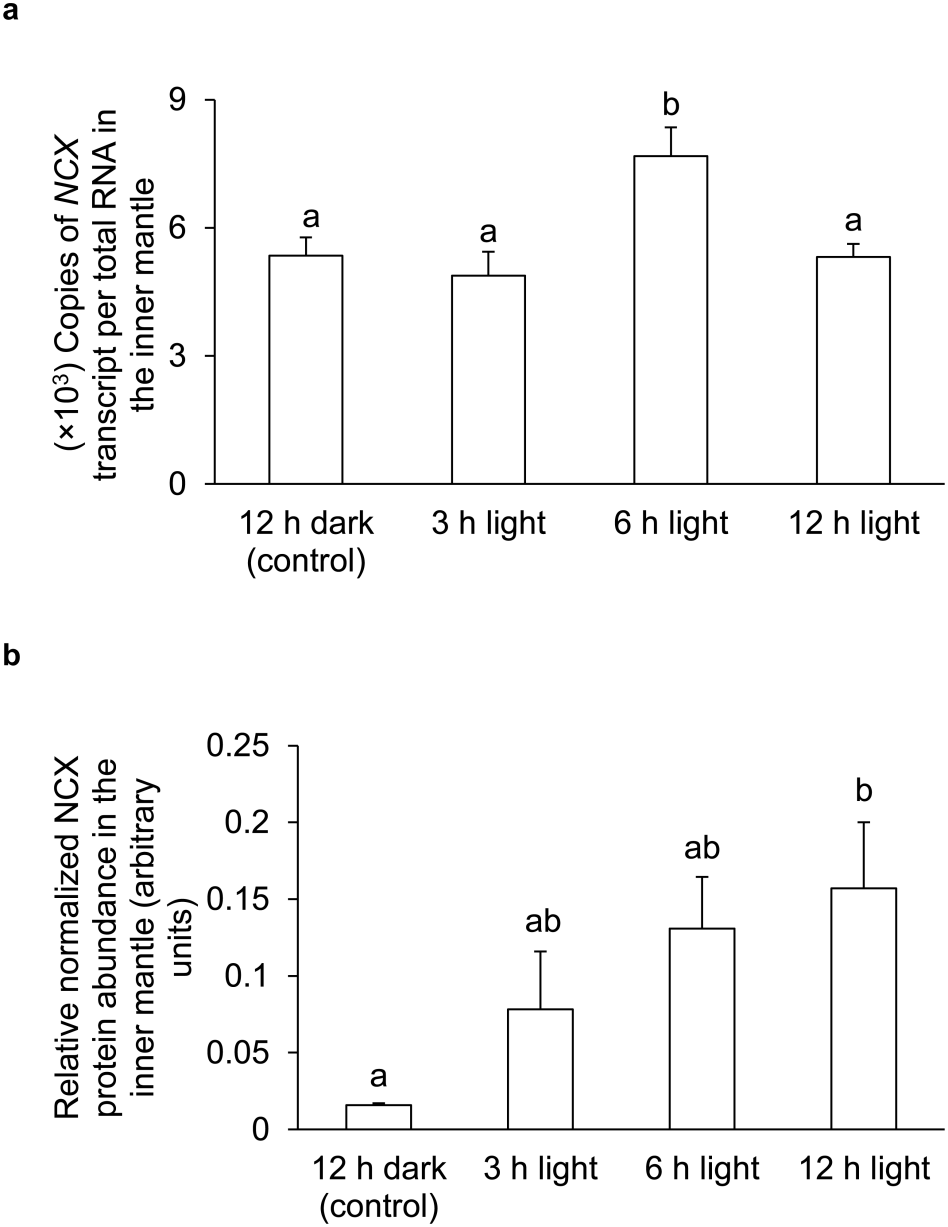
Effects of light on the mRNA expression level and protein abundance of *Na*^+^*/Ca*^*2+*^ *exchanger/* Na^+^/Ca^2+^ exchanger *(NCX*/NCX*)* in the inner mantle of *Tridacna squamosa*. (A) The transcript level (x 10^3^ copies of transcripts per ng total RNA) of *NCX* transcripts in the inner mantle of *T*. *squamosa* exposed to 12 h of darkness (control) or 3, 6 or 12 h of light. Results represent means + S.E.M (*N* = 4). Means not sharing the same letter are significantly different from each other (*P*<0.05). (B) The intensity of the NCX band for 50 μg protein was normalized with respect to that of tubulin. Results represent means + S.E.M (*N* = 4). Means not sharing the same letter are significantly different (*P*<0.05).

The transcript level of *NCX* increased significantly in the inner mantle of *T*. *squamosa* exposed to light for 6 h as compared with the control kept in darkness, although it returned back to the control level after 12 h of light exposure (Fig 8A). Furthermore, the protein abundance of NCX increased progressively the inner mantle of *T*. *squamosa* during light exposure, and became significantly greater than that of the control at hour 12 (Fig 8B).

## Discussion

### An overview of NKA and calcification

There is indirect evidence which suggests the involvement of NKA in biomineralization and calcification in animals. Before molting in terrestrial isopods, a large quantity of CaCO_3_ is stored between the epithelium and the old cuticle of the first four anterior sternites. The storage of CaCO_3_ results from the transport of large amounts of Ca^2+^ across the basolateral and apical membranes of the anterior sternal epithelium. In *P*. *scaber*, NKA is located at the basolateral membrane of the posterior and anterior sternal epithelium, where it could be involved indirectly in the transepithelial movement of Ca^2+^ [22]. The avian eggshell gland has a calcification process with unique circadian pattern, and comprises a tissue specialized in transporting the Ca^2+^ required for eggshell formation. During eggshell calcification, the gene expression of *NKAα* increases markedly in the avian eggshell gland, with a clear correlation between the level of *NKAα* expression and the stage of eggshell formation [23]. For corals (*A*. *yongei* and *Stylophora pistillata*), NKA is expressed in the basolateral membrane of calicoblastic cells [14]. Calcification in coral (*Galaxea fascicularis*) is inhibited by the Ca^2+^-ATPase inhibitor, ruthenium red, and by the NKA inhibitor, ouabain, indicating the involvement of active Ca^2+^ transport and Na^+^/Ca^2+^ exchange in the process [24]. Specifically, ouabain reduces the rate of light-enhanced calcification, but has no effect on the rate of calcification in darkness [24]. Hence, it can be concluded that NKA plays an indirect role in light-enhanced calcification in *G*. *fascicularis*. As for *T*. *squamosa*, we had obtained the full coding sequence of a *NKAα* from its inner mantle, which was localized predominantly to the shell-facing epithelium. We had also demonstrated that exposure to light upregulated the protein abundance of NKAα in the inner mantle and increased the anti-NKAα immunofluorescence of the shell-facing epithelium, denoting the possibility of its participation in light-enhanced calcification.

### Molecular characterization of NKAα from *T*. *squamosa*

NKAα comprises three Na^+^ and two K^+^ binding sites, as well as essential residues in the transmembrane domains, which coordinate the binding sites to release one type of cation and then bind to the other [28]. A comparison with the human NKAα [28] reveals that these coordinating residues are conserved in the NKAα of *T*. *squamosa*. Through mutational studies, Asp-742 in pig NKAα1 (corresponding to Asp-758 in *T*. *squamosa*) was shown to be a cytoplasmic K^+^ site [29] and this residue was well conserved in all the invertebrates NKAα aligned in Fig 1. This cytoplasmic K^+^ site was particularly important as it has been shown to stimulate the translocation of Na^+^ to the exterior [30–32]. The KETYY (Lys-Glu-Thr-Tyr-Tyr) motif is known for Na^+^ binding, and Na^+^ affinity is reduced by 96% when this motif was deleted from the C-terminus of NKAα [33]. Although the KETYY motif is present in the NKAα of all the invertebrates analyzed, its first amino acid varies between animal species. For *T*. *squamosa*, *Hydra vulgaris*, and *Schistosoma mansoni*, the first amino acid is lysine (i.e. KETYY). In *Crassostrea gigas* and *Ascaris suum*, the lysine residue is substituted by arginine (i.e. RETYY), while in *Doryteuthis opalescens*, it is replaced with asparagine (i.e. NETYY). This might indicate a variation in Na^+^ affinity among NKAα of mollusks, as demonstrated among *T*. *squamosa*, *C*. *gigas*, and *D*. *opalescens*.

All P-type ATPases including NKA contain the Asp-Lys-Thr-Gly-Thr (DKTGT) motif, of which the aspartate residue represents a phosphorylation site. This motif is conserved in NKAα of all the invertebrates examined, inclusive of *T*. *squamosa*. Two other motifs, Thr-Gly-Asp (TGD) and Thr-Gly-Asp-Gly-X-Asn-Asp (TGDGXND) are also conserved in NKAα of these invertebrates. They are involved in Mg^2+^ coordination associated with ATP binding at the phosphorylation site [34]. NKAα can be regulated by phosphorylation/dephosphorylation through cAMP-dependent PKA and PKC [35]. Ser-944 has been identified as a phosphorylation site for PKA in NKAα from the kidney of rat and the giant toad, *Bufo marinus* [36, 37]. This PKA phosphorylation site is conserved among all the NKAα of invertebrates, including *T*. *squamosa* (Ser-954). Site-directed mutagenesis of Nkaα from *B*. *marinus* indicates Thr-10 and Ser-11 as the cAMP-dependent PKC phosphorylation sites [36]. The NKAα of *H*. *vulgaris*, *S*. *mansoni*, and *A*. *suum* lack these PKC phosphorylation sites, but Thr-23 (corresponding to Thr-10 of *B*. *marinus*) is present in the NKAα of *T*. *squamosa*.

### The NKAα of *T*. *squamosa* can probably bind to NH_4_^+^ albeit with lower affinity than K^+^

It is apparent that the majority of invertebrate species, including *T*. *squamosa*, express only one form of NKAα. By contrast, multiple Nkaα isoforms have been identified in fish gills. For instance, the gills of the climbing perch, *A*. *testudineus*, typically express Nkaα1a, Nkaα1b, and Nkaα1c when exposed to fresh water, seawater and ammonia (in fresh water), respectively [38, 39]. Through mutation studies, Asn-786 of NKAα is known to be important for both Na^+^ and K^+^ binding [40]. While Asn-786 is present in Nkaα1c (the ammonia-isoform), it is replaced by Lys-786 in Nkaα1a (the freshwater-isoform) and Nkaα1b (the seawater-isoform) [38]. Similar to Nkaα1c of *A*. *testudineus* [38], the NKAα of *T*. *squamosa* consists of Asn-794 (equivalent to Asn-786 as mentioned by Pedersen et al. [40]) which is actually conserved among the invertebrate NKAα examined herein. Exposure of *A*. *testudineus* to ammonia leads to increases in the expression of *Nkaα1c*/Nkaα1c and the enzyme activity of NKA in its gills [38]. In addition, there are changes in the affinity of the branchial NKA to K^+^ and NH_4_^+^, with a greater increase in the K_m_ for NH_4_^+^ than for K^+^. Hence, it can be deduced that Nkaα1c can bind to both K^+^ and NH_4_^+^, with higher affinity to K^+^ than NH_4_^+^ [38]. Notably, the amino acids constituting the K^+^ binding sites in the NKAα of *T*. *squamosa* are identical to those in Nkaα1c, but different from those in Nkaα1a and Nkaα1b, of *A*. *testudineus* [38]. As expected, the NKA enzyme activity from the inner mantle of *T*. *squamosa* kept in darkness can be activated by either Na^+^/K^+^ or Na^+^/NH_4_^+^, and K^+^ has a higher efficiency of NKA activation than NH_4_^+^ [10]. Incidentally, the NKA from the gills of the blue crab, *Callinectes danae*, can be activated synergistically by K^+^ and NH_4_^+^ [41]. When NH_4_^+^ is included in a medium containing an optimized concentration of K^+^ to obtain close to *V*_max_ activity, there is a 90% increase in the NKA activity [41]. Hence, it has been proposed that the NKA of *C*. *danae* comprises two distinct K^+^ and NH_4_^+^ binding sites. However, molecular characterization of the NKA of *T*. *squamosa* did not reveal the two distinct types of binding site for K^+^ and NH_4_^+^. In crustaceans, active NH_4_^+^ excretion involves both NKA and V-type H^+^-ATPase [42,43], but bafilomycin-sensitive H^+^-ATPase activity is undetectable in the inner mantle of *T*. *squamosa* [10].

Exposure of *T*. *squamosa* to light leads to a significant increase in the Na^+^/NH_4_^+^-activated-NKA activity in the inner mantle, attributable to an increase in the effectiveness of NH_4_^+^ to replace K^+^ for NKA activation [10]. Indeed, our results confirm an increase in the protein abundance of the NKAα in the inner mantle of *T*. *squamosa* after 12 h of light exposure. However, they do not offer a satisfactory explanation on why a change in the affinity of NKA to K^+^ and NH_4_^+^ would occur. This is because, similar to many other invertebrates, the inner mantle of *T*. *squamosa* expressed only one form of NKAα. It has been proposed previously [10] that changes in the affinity of NKA to K^+^ and NH_4_^+^ in the inner mantle of *T*. *squamosa* could result from changes in expression of NKAβ, because NKAβ is known to alter the activity of NKA by modulating the affinity of NKAα for Na^+^ and K^+^ [44]. Therefore, efforts should be made in the future to determine the effect of light on *NKAβ*/NKAβ expression and its interaction with NKAα in the inner mantle of *T*. *squamosa*.

### NKAα is localized mainly to the basolateral membrane of the shell-facing epithelium

In agreement with immunocytochemical and histochemical localization of basolateral NKA in non-excitable epithelial tissues of insects [45, 46], crustaceans [47, 48], teleosts [49] and mammals [50, 51], NKAα was localized ubiquitously to the basolateral membrane of the shell-facing epithelium of the inner mantle of *T*. *squamosa*. This asymmetric distribution of NKA in epithelial cells facilitates and determines the vectorial transepithelial transport of water and certain ions across the epithelium [52]. Notably, NKAα- immunofluorescence was weak along the seawater-facing epithelium while faint and scattered among the loose connective tissues of the inner mantle. Therefore, it is logical to deduce that the basolateral NKAα of the shell-facing epithelium, which is in direct contact with the extrapallial fluid, plays a certain role in shell formation in *T*. *squamosa*. However, with its basolateral localization, it is unlikely to be involved in the uptake of H^+^ as NH_4_^+^ in the extrapallial fluid by the shell-facing epithelial cells as suggested previously [10], and the possible involvement of other types of ammonia transporters in the process should be considered.

### Light exposure increased the protein abundance of NKAα in the inner mantle

After 3 h of exposure to light, there was a transient decrease in the transcript level of *NKAα* in the inner mantle of *T*. *squamosa*, but the protein abundance of NKAα remained unchanged as compared to the control in darkness. In fact, the NKAα protein abundance increased progressively from hour 3 onwards and became significantly greater than the control value at the 12^th^ h of light exposure. The reason for the transient decrease in *NKAα* transcript level is unclear at present, but it would have minimal physiological significance due to the unchanged NKAα protein abundance. The significant increase in protein abundance of NKAα after 12 h of light exposure could be related to an upregulation of its production through increased translation or a downregulation of its degradation. Either way, it can be concluded that NKAα, and hence NKA activity, was regulated mainly at the protein level. A role for the NKA in light-enhanced calcification in *T*. *squamosa* is further supported by a significantly stronger basolateral NKAα-immunofluoresence of the shell-facing epithelium of clams exposed to light for 12 h. It is probable that more NKAα was needed in the shell-facing epithelial cells of the inner mantle to balance a rise in Na^+^ influx, possibly caused by increases in activities of some Na^+^-dependent ion transporters/channels involved in light-enhanced calcification.

### Light-enhanced expressions of enzymes/transporters in *T*. *squamosa* and their implications

Besides NKA, three other transporter and enzyme also display light-dependent expression in *T*. *squamosa*. Giant clams are known to absorb and assimilate ammonia from the external medium during insolation [53, 54]. Recently, Hiong et al. [25] have reported that the transcript level and protein abundance of a host Glutamine Synthetase, which assimilates NH_4_^+^ into glutamine, in the ctenidium of *T*. *squamosa*, are up-regulated by light exposure. Light also enhances the transcript level and protein abundance of a Na^+^/H^+^ exchanger 3-like transporter, which mediates H^+^ efflux in exchange for Na^+^ uptake in a 1:1 stoichiometry, in the ctenidium, indicating that it may be involved in increased H^+^ excretion in pursuance of whole-body acid-base balance during light-enhanced calcification [26]. In addition, light exposure leads to significant increases in the transcript level and protein abundance of Plasma Membrane Ca^2+^-ATPase (PMCA) in the inner mantle of *T*. *squamosa* [55]. As PMCA is localized predominantly to the apical membrane of the shell-facing epithelial cells of the inner mantle, it offers insight into a light-dependable mechanism of shell formation in *T*. *squamosa* and a novel explanation of light-enhanced calcification in general [55]. Cohen et al. [56] reported recently that calcification in corals *Porites lutea* and *Acropora variabilis* were mostly enhanced by blue light, but photosynthesis was less efficient under that part of the spectrum. They suggested that blue light photoreceptors in coral tissue could be the light sensor which activated a plasma membrane Ca^2+^-ATPase (PMCA) involved in light-enhanced calcification [56]. In view of the light-dependent expression of genes and proteins in *T*. *squamosa*, it would be rewarding to examine whether scleractinian corals would display similar light-dependent phenomena.

As transcription and translation are energy-dependent processes, daily changes in gene and protein expression levels in *T*. *squamosa* appear to be energy-wasteful [25]. However, giant clams live in symbiosis with zooxanthellae which translocate 90–95% of the carbon fixed daily during photosynthesis to the host clam [57–59]. The compounds translocated to the host include glycerol, glucose, and amino acids [60, 61] and the quantity of translocated carbon is sufficient to meet the daily energy and growth requirements of the clam [2, 62–64]. Probably because of that, *T*. *squamosa* can afford energetically to regulate light-dependent processes through transcriptional and translational changes. Light can be detected by the siphonal eyes located at the surface of the host’s hypertrophied siphon [65], which transmit signals to other parts of the body. However, whether the transmission process involves hormonal or neuronal signals is uncertain at present. It is also possible that light can be detected by the photoreceptors of *Symbiodinium* which are known to possess rhodopsin [66]. It has been proposed that, in response to light, the symbionts may release some sort of signaling molecules which can augment the transcription and/or translation of certain genes/proteins in the host as proposed previously [10, 25, 26]. Thus, in order to examine the effects of symbionts on the host under light exposure, future work should involve the elimination of the symbionts from the host.

### The possible relationships between the transmembrane Na^+^ gradient and calcification in *T*. *squamosa*

Shell formation in *T*. *squamosa* requires the supply of Ca^2+^ and HCO_3_^−^ from the hemolymph to the extrapallial fluid through the shell-facing epithelium of the inner mantle, and the removal of H^+^ in the opposite direction. Many transporters and channels systems located along the apical and basolateral membranes of these epithelial cells could be involved in generating the transepithelial ion fluxes. Given that it is capable of vectorial transport, the distribution of the transporters between the apical and basolateral membranes of the shell-facing epithelium must be asymmetrical. Sano et al. reported that the shell of *Tridacna derasa* displayed diurnal variations of Sr/Ca ratio [8]. Their results suggested the involvement of an apical Ca^2+^-ATPase which would selectively incorporate Ca^2+^ at high-calcification rates during light-enhanced calcification, and result in widened daytime growth band with reduced Sr/Ca ratio in the giant clam shell. Besides Ca^2+^-ATPase, other transporters like NCX (SLC8) [24], bicarbonate anion transporters (BATs; SLC4 and SLC26) [14, 67–69] and NHE (SLC9) may also participate in the calcification process, and the operation of these transporters relies on the favorable Na^+^ gradient generated by the basolateral NKA. Indeed, we obtained results which confirm that the gene and protein expression levels of *NCX*/NCX in the inner mantle are also light-dependent. As NCX employs the Na^+^ gradient to transport 3 Na^+^ in and 1 Ca^2+^ out of the cell, an increase in NKA activity is required to maintain the intracellular Na^+^ homeostasis of these epithelial cells in the shell-facing epithelium. Hence, efforts should be made in the future to elucidate the functional roles of NCX in light-enhanced calcification in the inner mantle of *T*. *squamosa*.

### Perspective

NKAα has a basolateral localization in the shell-facing epithelium of the inner mantle in *T*. *squamosa*. Exposure to light for 12 h leads to a significantly increase in protein abundance of NKAα in the inner mantle and the basolateral NKAα-immunofluorescence along its shell-facing epithelium, indicating that NKA may play an indirect role in light-enhanced calcification. These results provide indirect evidence on the involvement of Na^+^-coupled transporters in light-enhanced calcification, and a firm basis for further investigation on vectorial Na^+^-dependent ion transport across the shell-facing epithelium of the inner mantle. It is probable that many of the transporters involved in the calcification process are expressed differentially between the apical and basolateral membranes. However, it is unlikely that every epithelial cell would express exactly the same transporters and perform identical ion transport functions. Hence, future experiments should aim to elucidate membrane-specific transport systems and different cell types involved in the vectorial transport of various ions in the shell-facing of the inner mantle in order to fully understand the mechanisms of light-enhanced calcification in *T*. *squamosa*.
